# Full endoscopic resection of ventral thoracic osteophyte and repair of spontaneous CSF leak

**DOI:** 10.3171/2024.1.FOCVID23209

**Published:** 2024-04-01

**Authors:** Nelson Sofoluke, Jannik Leyendecker, Christoph P. Hofstetter, Sanjay Konakondla

**Affiliations:** 1 Department of Neurosurgery, Geisinger Neuroscience Institute, Danville, Pennsylvania;; 2 Department of Neurological Surgery, University of Washington, Seattle, Washington; and; 3 Department of Orthopedics and Trauma Surgery, University of Cologne, Faculty of Medicine, Cologne, Germany

**Keywords:** full endoscopic spine surgery, CSF leak, repair

## Abstract

Spontaneous CSF leaks frequently cause headaches, meningismus, and nausea due to intracranial hypotension. When conservative treatment fails, surgical repair is indicated. Especially ventral leaks necessitate invasive approaches with substantial blood loss and tissue trauma. Full endoscopic spine surgery (FESS) enables circumferential access via the transforaminal approach. Here, the authors show the successful repair of a ventral CSF leak in the thoracic spine after removal of bony osteophytes utilizing FESS with placement of a dural substitute and sealant. Lasting symptom relief was reported. These results suggest that FESS is safe and efficient for the repair of spontaneous and incidental CSF leaks.

The video can be found here: https://stream.cadmore.media/r10.3171/2024.1.FOCVID23209

## Transcript

This case demonstrates the application of full endoscopic uniportal spine techniques to safely remove a disc osteophyte complex and to repair a spontaneous cerebrospinal fluid leak in the thoracic spine. I’m Sanjay Konakondla and I’ll review the patient workup, technical nuances, and intraoperative considerations we made for a successful surgical result.

### 0:42 Patient Presentation.

The patient is a 43-year-old female with a past medical history significant for anxiety and depression who suffered from multiple years of long-standing headaches, dizziness, double vision, and generalized malaise. Previous workup identified tonsillar ectopia. However, the clinical workup and questioning revealed symptoms more closely related to intracranial hypotension. She had significant exacerbation of headaches with any amount of activity, which included sitting up from a supine position or standing from a seated position.^1,2^ She also failed conservative management, which also included empiric epidural blood patch.

### 1:18 Imaging Review.

Imaging available for review included an MRI of the brain as well as the cervical spine, which revealed a full sella and findings suggestive of intracranial hypotension. The cervical MRI revealed cerebellar ectopia as well as extradural cerebrospinal fluid in the high thoracic spine. Thoracic spine MRI revealed a significant T7–8 disc osteophyte complex with ventral compression of the spinal cord and findings of extradural cerebrospinal fluid. A prone position CT myelogram revealed a fast cerebrospinal fluid leak at the level of the T7–8 disc osteophyte complex. The patient was deemed appropriate for an operative intervention for disc osteophyte removal and repair of the spinal fluid leak.^3^

### 2:05 Patient Positioning/OR Setup.

A full endoscopic surgical procedure was chosen to optimize intraoperative visualization while minimizing the surgical invasiveness, and to avoid an open intradural approach, which may necessitate greater spinal cord manipulation. After full informed consent the patient was scheduled electively for this procedure. Intraoperative positioning included an open Jackson table with a Wilson frame. The patient was induced under general endotracheal anesthesia without complication and positioned prone onto the operating table. All pressure points were accounted for and padded. Intraoperative neuromonitoring was used throughout the case. Screens were opposite the surgeon for comfort. Surgery was completed from a right-sided approach as the leak was slightly eccentric to the right side.^4,5^

### 2:49 Targeting and Docking.

Intraoperative fluoroscopy is used to identify specific landmarks. After the appropriate level is identified, lines are drawn at the midline and at the disc level. The C-arm on the AP view was used to mark the appropriate level, and a spinal needle was placed on the caudal pedicle of the contralateral side. This static marker at the level of interest is our preferred technique when completing endoscopic approaches in the thoracic spine to create a reproducible and constant point of reference throughout the procedure. A spinal needle is advanced into Kambin’s triangle. A K-wire is placed through the spinal needle, the spinal needle is removed, and sequential dilators are placed over the K-wire. A tubular retractor is then advanced over the final dilators. The system used in this case was the joimax TESSYS set, which has a tubular retractor inner and outer diameter of 6.5 mm and 7.5 mm, respectively. This allows for the passage of an endoscope with a 3.7-mm working channel diameter and facilitates Kerrison instruments up to 3.5 mm and a round diamond drill of 3.5 mm in diameter.

### 4:03 Introduction of the Endoscope.

The endoscope is introduced into the tubular retractor, and a high-speed diamond tip drill is used to remove the partial inferior articulating process of T7 and partial superior articulating process of T8. After minimal drilling the joint space can be identified as is labeled by the still image. Once the joint space is identified, further drilling can be completed through the superior articulating process of T8 to enter the epidural space.

### 4:32 First Entrance Into Epidural Space.

Once the epidural space is identified, drilling is continued to enlarge the opening to access the disc space and the epidural space. Epidural veins are identified once the medial superior articulating process is removed, and the distinction between the dura and the epidural veins and fibers of the epidural space is critical, and we stress the practice of "trusting the fat" to continue safely to identify the spinal cord dura. Pituitary graspers are used to clear the epidural space and expose the spinal cord dura. This is where it is absolutely necessary to properly identify tissue planes.

### 5:24 Disc Identification and Creating Space Ventral to the Spinal Cord.

The disc herniation can be identified ventral to the spinal cord as shown in the still image. To facilitate safe entry ventral to the spinal cord, a high-speed drill is used to remove part of the dorsal vertebral body of T7 and T8 and further disc resection is completed. We recommend our vertebral body core technique to not only easily identify anatomy, but to avoid spinal cord manipulation. This also keeps a barrier between the drill bit at the spinal cord dura. The disc space is then followed medially to identify any further disc herniations and to facilitate visualization directly ventral to spinal cord dura. A curved ball-tipped bipolar device can be passed ventral to the spinal cord dura and dorsal to the disc herniation. Additional disc herniation can be appreciated as we have the vertebral bodies appropriately and clearly identified. This is freed from the ventral dura and carefully removed with pituitary graspers both straight and slightly up-angled. It is important to note here that the direction of release should always be directed ventrally into the space created and away from the spinal cord. This technique ensures the avoidance of spinal cord trauma when removing discs through an angled scope view.

### 7:05 Spinal Cord Decompressed and Identification of Dural Defect.

After removal of the final disc components, the spinal cord is now decompressed. Attention can now be placed on identification of the ventral dural defect. A curved instrument was used to identify this defect, which is shown with the blue arrow in the still image. Once identified, the defect was inspected thoroughly.

### 7:30 Repair of Dural Defect.

Small pieces of dural substitute were cut and used for repair of the dural defect. Dural substitute was placed as an inlay followed by an onlay of various dural substitute pieces and sizes. During these maneuvers the continuous irrigation was stopped intermittently to ensure proper placement of the dural substitute. The laxity of the dura can be appreciated here due to the extradural spinal fluid leak. A dural sealant was subsequently applied. The scope and the tubular retractor were removed, and the incision was closed with absorbable sutures and glue.^6^ Surgery time was 148 minutes, and estimated blood loss was about 10 cc.

### 8:20 Postoperative Course.

The patient was admitted to the hospital and observed closely. She was laid flat overnight and gradually mobilized on postoperative day 1 to postoperative day 3 and was discharged on postoperative day 3, reporting immediate symptom relief. The patient was followed closely for rebound headaches, and a CT of the head was completed, which revealed stable ventricle size. Postoperative MRI of the thoracic spine revealed a decompressed spinal cord with removal of the disc osteophyte complex and significant decrease in extradural cerebrospinal fluid. It should be noted that these cases are challenging, and specific consideration should be given to preoperative planning. Comprehensive training in full endoscopic spine procedures, clinical experience, and surgical volume of endoscopic spine procedures are necessary for successful surgical outcomes.

## Disclosures

Dr. Hofstetter reported consulting fees from Globus, Innovasis, and joimax. He also teaches for AO Spine and Espinea. Dr. Konakondla reported educational consulting fees and royalties from Spineology and educational consulting fees and honoraria from Stryker, joimax, and Elliquence.

## Author Contributions

Primary surgeon: Konakondla. Assistant surgeon: Sofoluke. Editing and drafting the video and abstract: Konakondla, Sofoluke, Leyendecker. Critically revising the work: all authors. Reviewed submitted version of the work: Konakondla, Leyendecker, Hofstetter. Approved the final version of the work on behalf of all authors: Konakondla. Supervision: Konakondla, Hofstetter.

## Supplemental Information

### Patient Informed Consent

The necessary patient informed consent was obtained in this study.

## References

[1] Schievink WI Maya MM Jean-Pierre S Nuño M Prasad RS Moser FG A classification system of spontaneous spinal CSF leaks Neurology 2016 87 7 673 679 27440149 10.1212/WNL.0000000000002986

[2] Jones MR Shlobin NA Dahdaleh NS Spontaneous spinal cerebrospinal fluid leak: review and management algorithm World Neurosurg 2021 150 133 139 33798778 10.1016/j.wneu.2021.03.115

[3] Thielen KR Sillery JC Morris JM Ultrafast dynamic computed tomography myelography for the precise identification of high-flow cerebrospinal fluid leaks caused by spiculated spinal osteophytes J Neurosurg Spine 2015 22 3 324 331 25555057 10.3171/2014.10.SPINE14209

[4] Oertel JM Burkhardt BW Full endoscopic treatment of dural tears in lumbar spine surgery Eur Spine J 2017 26 10 2496 2503 28528480 10.1007/s00586-017-5105-8

[5] Barber SM Sofoluke N Reardon T Full endoscopic repair of spontaneous ventral cerebrospinal fluid leaks in the spine: systematic review of surgical treatment options and illustrative case World Neurosurg 2022 168 e578 e586 36243360 10.1016/j.wneu.2022.10.039

[6] Sencakova D Mokri B McClelland RL The efficacy of epidural blood patch in spontaneous CSF leaks Neurology 2001 57 10 1921 1923 11723293 10.1212/wnl.57.10.1921

